# Peanut thresholds in peanut‐allergic children are related to dietary composition

**DOI:** 10.1002/iid3.841

**Published:** 2023-05-26

**Authors:** Daisy Luiten, Maarten Biezeveld, Olga van Doorn, Hanae Riady, Ming Yang, Femke Bergsma, Atie van der Plas, Kim Brand, Nicolette Arends, Annette de Bruin, de Vries, Tim de Meij, Berber Vlieg‐Boerstra

**Affiliations:** ^1^ Department of Paediatrics OLVG Hospital Amsterdam The Netherlands; ^2^ Department of Pediatric Pulmonology and Allergology Amsterdam University Medical Centers Amsterdam The Netherlands; ^3^ Department of Pediatric Pulmonology and Allergology Erasmus MC Sophia Children's Hospital Rotterdam The Netherlands; ^4^ Department of Paediatrics Tergooi Hospital Blaricum The Netherlands; ^5^ Division of Human Nutrition and Health Wageningen University Wageningen The Netherlands; ^6^ Department of Paediatric Gastroenterology Amsterdam University Medical Centers Amsterdam The Netherlands

**Keywords:** diet, microbiome, oral food challenge, peanut allergy, thresholds

## Abstract

**Background:**

There is no clear explanation for the large variation in threshold levels among peanut‐allergic children. We hypothesized that diet composition can partly explain this variation in thresholds, as nutrients and foods influence the intestinal barrier function and microbiota.

**Aim:**

to explore the relationship between the threshold levels for peanut and nutritional intake and gut microbial composition in peanut‐allergic children.

**Methods:**

In this explorative cross‐sectional study the cumulative threshold levels for peanut were determined by oral food challenge tests. Data on nutrients and foods consumed were obtained from 3‐day food diaries. Microbial composition of faeces and saliva were determined by molecular microbiota detection technique. Multivariable linear regression analysis and multiple logistic regression were used to explore the associations, adjusted for energy and senitization.

**Results:**

Sixty‐five children were included, of whom 32 (49%) (median age 50 months, IQR 28.0–96.5) had a positive oral food challenge. Significant positive associations were found between the intake of total carbohydrates, vitamin A and cumulative threshold levels for peanut, while significant negative associations were found for long‐chain polyunsaturated fatty acids, linoleic acid and omega‐6 fatty acids. No associations were found between threshold levels and microbial composition of faeces and saliva. However, a significant higher abundance of Proteobacteria and Bacteroidetes in saliva (*p* = 0.011 and 0.04, respectively) and of Proteobacteria in faeces (*p* = 0.003) were found in children with a positive peanut challenge compared to children with a negative peanut challenge.

**Conclusion:**

As a novel concept, this study showed that dietary composition is related to threshold levels for peanut.

## 1 INTRODUCTION

A threshold level is defined as the lowest (cumulative) amount of peanut causing an allergic reaction.^1^, ^2^ Threshold levels for peanut, as determined in Open Food Challenges (OFCs) or Double‐Blind, Placebo‐Controlled Food Challenges (DBPCFCs), may largely vary from milligrams to grams in peanut‐allergic children.^1^ A systematic review and meta‐analysis in over 3000 patients showed that 4.5% (95% CI, 1.9%–10.1%) of individuals reacted to 5 mg or less of peanut protein with anaphylaxis.^2^

There is no clear explanation for these large differences in observed threshold levels in oral food challenges. Several cofactors are known to influence threshold levels in accidental food exposure. However, most of these are not relevant in children, such as intake of alcohol and NSAIDs, or they do not play a role in controlled oral food challenges settings, such as exercise, viral infections and sleep deprivation.^3^

An explanation for the variation in thresholds for peanut could be an impaired intestinal barrier function and subsequently an increased intestinal permeability, allowing enhanced uptake of allergens. This is in agreement to the recently launched novel extended “epithelial barrier hypothesis,” in which it is proposed that a defective epithelial barrier is linked to the increased use of damaging agents due to industrialization, urbanization and modern life, such as the use of processed foods.^4^ Mucosal barrier dysfunction has been demonstrated in food allergy, asthma, chronic rhinosinusitis, atopic dermatitis, and Eosinophilic Esophagitis.^5^, ^6^

In turn, various nutrients and food compounds are essential for the maintenance of this intestinal barrier function and are also important for the intestinal microbiota. For example, short‐chain fatty acids (SCFAs) produced through microbial fermentation of dietary fiber maintain mucosal integrity by processes including mucus production, tissue repair and upregulation of the expression of tight junction proteins.^7^, ^8^ In addition, vitamin A plays a critical role in the differentiation of cells toward mucosal barrier function.^9^ Zinc and iron have an essential role in the maintenance of intestinal epithelial tight junction barrier via the regulation of claudin‐3 and occluding expression.^10^, ^11^ Vitamin D may have an effect on allergen sensitivity, because of the involvement in the maintenance of the intestinal barrier function and several mechanisms that promote immunologic tolerance.^12^ In contrast, there is abundant evidence that a Western diet rich in (saturated) fat, meat, refined sugars and processed foods is related to low‐grade inflammation, disturbed barrier function and pro‐inflammatory microbiome composition.^13^, ^14^

Therefore, we hypothesized that the composition of the diet can partly explain the variation in threshold levels for peanut in peanut‐allergic children, as nutrients and foods both influence the intestinal barrier function and intestinal microbiota.

In this explorative study we aimed to analyse the association between the threshold levels for peanut and intake of nutrients and foods as well as the gut and oral microbial composition in peanut‐allergic children.

## 2 METHODS

### 2.1 Study design and participants

This explorative cross‐sectional study was performed between May 2017 and May 2020 in two tertiary hospitals (Erasmus MC Sophia Children's Hospital and Amsterdam UMC) and two secondary hospitals (OLVG hospital and Tergooi hospital) in the Netherlands. Children in the age of 0–18 years with a suspected peanut allergy in whom an oral peanut challenge was planned for initial diagnosis or follow‐up evaluation of their peanut allergy, were approached for participation in this study. Children were included based on history and peanut sensitization (peanut sIgE ≥0.35 kUA/L (Thermo Fisher) or peanut skin prick test (SPT) HEP‐index area of ≥0.4 (ALK‐Abelló)), determined no longer than 1 year before the peanut challenge.^15^ Medical ethical approval for the study was obtained by the Medical Ethical Committee of the OLVG hospital, nr. WO17.000.

### 2.2 Peanut food challenge

OFC and DBPCCFCs were performed according to the PRACTALL‐consensus protocol with dosages ranging from 3 to 3000 mg of peanut protein using a semilogarithmic increase (3‐10‐30‐100‐300‐1000‐3000 mg peanut protein).^16^ In this study, an extra low starting dose of 1 mg of peanut protein was added to the incremental scale. Symptom severity was scored as 0 (none), 1 (mild), 2 (moderate), or 3 (severe), according to PRACTALL. The child was considered peanut‐allergic and the challenge was stopped, when there was an objective reaction to peanut or when there was a subjective reaction to at least three subsequent doses of peanut during the OFC or DBPCFC. The cumulative dose of peanut (Maximal 1‐4‐14‐44‐144‐444‐1444‐4444 mg) administered during the challenge was considered as the threshold dose.^16^

### 2.3 Dietary intake analysis

Dietary intake of energy, macronutrients, micronutrients, foods and food groups were assessed using 3‐day semistructured food diaries (1 weekend day and 2 week days), completed within 7 days before or after the peanut challenge, by caregivers or by the children themselves when older than 11 years. We chose for this method because this method is accepted as a representative for the usual intake and as it was shown a feasible method in European children.^17^ We asked to maintain the usual diet during the study, to avoid great changes in dietary intake. Only extreme dietary changes will alter the human microbiome in just a few days.^18^, ^19^ Patients were asked to record the intake of food in grams or household portion sizes, as well as to record in detail types and brands of the consumed foods. All consumed foods and drinks were coded and entered by the dietitians in the online‐nutrition calculation program Compleat (Wageningen University, the Netherlands), based on the Dutch food composition database NEVO 2016.^20^, ^21^ Average intake of energy, nutrients (with and without supplements) and foods were calculated. Additionally, all consumed foods (grams/day) were allocated into food groups (Supporting Information: Tables 1 and 2).

As our study population consisted of children from different age groups with varying energy needs dietary intake was energy‐adjusted (calculation per 1000 kcal).

In addition, patients' intake of docosahexaenoic acid (DHA) and eicosapentaenoic acid (EPA) was assessed by a self‐compiled 6‐item Food Frequency Questionnaire (FFQ) on lean, semifat, fatty fish and shellfish. For omega‐6 (n‐6) long‐chain polyunsaturated fatty acids (LCPUFAs) the ratio of omega‐6 to omega‐3 (n‐3) LCPUFAs (n‐6:n‐3 ratio) was used.^22^

### 2.4 Saliva and faeces microbiota characterization

Saliva samples were collected by a nurse by oral swab on the day of the food challenge, before the start of the challenge and were immediately frozen at –20°C. Faeces samples were obtained by the parents within 2 days before the challenges. Samples were collected using eNAT **®** (Copan) sterile tubes which contained a detergent and a protein denaturant that prevents microbial proliferation and ensures optimal preservation of RNA and DNA at room temperature up to 4 weeks.^23^ Samples were stored at OLVG hospital at –20°C until microbial analysis.

#### 2.4.1 Sampling preparation, DNA extraction, and polymerase chain reaction

All faeces and saliva samples were prepared and analyzed by the standard IS‐pro procedure.^24^ IS‐pro is a DNA‐based microbiota profiling technique, based on the identification of species‐specific length polymorphisms of the interspacer (IS) region and phylum‐specific sequence polymorphisms of 16S rDNA. Consequently, IS‐pro generate data on both species and phylum level. Relative abundance of Firmicutes, Actinobacteria, Fusobacteria, Verrucomicrobia (FAFV), Bacteroidetes and Proteobacteria was obtained, measured in relative fluorescence units (RFU). Detailed information is given in Supporting Information: Appendix 1.

### 2.5 Statistical methods

As this was an explorative study and to the best of our knowledge no similar previous studies were performed, no power analysis was performed.

Multiple linear regression was used to explore the associations between the average dietary intake of nutrients, foods and food groups per 1000 kcal and the threshold levels for peanut. Specific IgE level and SPT were classified as weak positive (sIgE 0.35–0.7 kU/l or HEP‐index 0.4–0.8), positive (sIgE 0.7–17.5 kU/l or HEP‐index 0.8–1.1) and strong positive (sIgE >17.5 kU/l or HEP‐index >1.1) and were added as potential confounder.^25^

Multiple logistic regression was used to explore the associations between the faecal and saliva microbiota composition at phylum level between children having the lowest 50% of thresholds (<445 mg cumulative peanut protein) and children with the highest 50% threshold levels for peanut (≥445 mg cumulative peanut protein).

Mann–Whitney *U* tests or two‐sample *T*‐tests were used to test differences in composition and diversity of the microbiota between children with a positive and negative food challenge.

IBM SPSS Statistics versions 25 (SPSS Inc) was used for all statistical analysis.

## 3 RESULTS

### 3.1 Characteristics of study population

In total 65 children were included. Two children were excluded because they did not fill in their food diary. Thirty‐two children (49%) (median age 50 months, IQR 28.0–96.5) had a positive OFC or DBPCFC and were classified as peanut‐allergic. Thirty‐three children (51%) (median age 63 months, IQR 33.0–99.5) had a negative OFC or DBPCFC and were classified as tolerant for peanut. Patient characteristics of the included children are presented in Table 1. SPT and Ara h 2 were significantly higher in the allergic compared to the tolerant children, although Ara h 2 was determined in only 27/65 children. These data showed that 51% of children were sensitized without clinical relevance to peanut. Children with a negative challenge test had significantly more often attended day care.

**Table 1:** Patients characteristics.

	Total (*n* = 65)	Allergic (*n* = 32)	Tolerant (*n* = 33)	*p* Value
Gender (male), *n* (%)	45 (69.2)	22 (68.8)	23 (69.7)	0.934
Age in months, median (IQR)	56 (31.0–98.0)	50 (28.0–96.5)	63 (33.0–99.5)	0.420
Current other atopic diseases, *n* (%)	61 (93.9)	30 (93.8)	31 (93.9)	1.000
Atopic dermatitis	59 (90.8)	30 (93.8)	29 (87.9)	0.672
Asthma (like symptoms)	19 (29.2)	12 (37.5)	7 (21.2)	0.149
Hay fever	21 (32.8)	12 (38.7)	9 (27.3)	0.417
SIgE for peanut (kU/L), median (IQR)	1.67 (0.70–6.65)	1.82 (0.74–11.60)	1.17 (0.65–3.11)	0.187
	(*n* = 45)	(*n* = 27)	(*n* = 18)	
Ara h2 (kU/L), median (IQR)	1.24 (0.59–4.24)	3.02 (1.32–12.06)	0.59 (0.0–0.98)	**0.001** *
	n = 27	n = 14	n = 13	
SPT peanut HEP‐index area, median (IQR)	1.2 (0.8–2.13)	2.13 (1.63–2.5)	0.96 (0.77–1.61)	**0.033** *
	*n* = 23	*n* = 7	*n* = 16	
Reaction to peanut in history, *n* (%)				
Yes reaction	25 (38.5)	13 (40.6)	12 (36.3)	0.793
Unknown	5 (7.7)	2 (6.3)	3 (9.1)	
First atopic symptoms, age in months, median (IQR)	3 (1.0–6.0)	3 (1.0–6.0)	4 (2.0–6.75)	0.334
	*n* = 63	*n* = 31	*n* = 32	
Cesarean delivery, *n* (%)	8 (12.9	2 (6.7)	6 (18.2)	0.170
Antibiotic used in 0–3 years, *n* (%)				
Unknown	20 (30.8)	12 (37.8)	10 (31.2)	n.a.
Yes (used)	23 (35,3%)	11 (34,3%)	12 (36,3%)	0.537
Day care attendance, *n* (%)	59 (90.8)	26 (81.3)	33 (100)	**0.011** *
Pets currently at home, *n* (%)	13 (20)	7 (21.9)	6 (18.2)	0.710
Siblings, *n* (%)	51 (78.5)	27 (84.4)	24 (72.7)	0.253

Abbreviations: HEP, histamine equivalent prick; IQR, inter quartile range; sIgE, allergen‐specific immunoglobulin E; SPT, Skin Prick Test.

* Significantly related to the outcome, *p* < .05.

### 3.2 Food challenge outcome

Thirty‐two (49%) were positive. Of these, 19 were performed by OFC and 13 by DBPCFC. The median cumulative threshold level was 456 (IQR 144–1444) mg of peanut protein. No reactions were observed to the first dose of 1 mg peanut protein. The most common threshold level (28.1%) was dose 7 (1444 mg cumulative peanut protein). In five allergic children the last challenge dose was repeated and in three children the last dose was not totally consumed due to an allergic reaction. In Table 2 the distribution of the (cumulative) threshold level is depicted. Thirty‐one out of 32 (96.9%) allergic children had an objective reaction.

**Table 2:** Distribution of the (cumulative) threshold dose with peanut in oral food challenges.

	Dose	Cumulative threshold dose peanut protein^a^	No (%) of children	PRACTALL severity score		
				No (%) of children, *N* = 32		
			*N* = 32			
				Score 1	Score 2	Score 3
				*N* = 14 (43.8%)	*N* = 14 (43.8%)	*N* = 4 (12.5%)
Dose 1	1 mg	1 mg	0	0	0	0
Dose 2	3 mg	4 mg	3 (9.4%)	3 (9.4%)	0	0
Dose 3	10 mg	14–24 mg	3 (9.4%)	2 (6.3%)	1 (3.1%)	0
Dose 4	30 mg	44 mg	1 (3.1%)	1 (3.1%)		
Dose 5	100 mg	144–194 mg	5 (15.6%)	1 (3.1%)	2 (6.3%)	2 (6.3%)
Dose 6	300 mg	442–744 mg	8 (25.0%)	1 (3.1%)	5 (15.6%)	2 (6.3%)
Dose 7	1000 mg	1444–1744 mg	9 (28.18%)	4 (12.5%)	5 (15.6%)	0
Dose 8	3000 mg	4169–4444 mg	3 (9.4%)	2 (9.4%)	1 (3.1%)	0

Abbreviation: Mg, milligram.

^a^ Reported cumulative doses could fall within a range, due to repeated doses or allergic reactions before the whole dose was ingested.

Fourteen out of the 32 allergic children (43.8%) had a maximum PRACTALL score of 1 as severity, 14 children (43.8%) had a maximum score of 2, and 4 children (12.5%) had a maximum PRACTALL severity score of 3. In Table 2 the distribution of the PRACTALL severity score is presented.

### 3.3 Associations between nutrients, foods, and threshold level

Three‐day food diaries were collected in all children. Foods were allocated to 39 food groups. A significant positive association was found between the intake of total carbohydrates (*β* = .478, *p* = 0.011), vitamin A expressed in retinol activity equivalents (RAE) (*β* = .552, *p* = 0.001) and round toast/crackers (*β* = .556, *p* = 0.001), all adjusted for energy intake and sensitization (Table 3). A significant negative association was found for LCPUFAS (*β* = –.470, *p* = 0.0015), linoleic acid (*β* = –.389, *p* = 0.034) and omega‐6 fatty acids from food (*β* = –.385, *p* = 0.038).

**Table 3:** Associations in peanut‐allergic children (*n* = 32) assessed by multivariable linear regression analyses between intake of nutrients and foods and cumulative threshold levels in milligram peanut protein adjusted for senitization.

	Standardized *β* Coefficients (95% CI), adjusted for sIgE and SPT categories	Adjusted *R* ^2^	*p* Value
*Nutrients per 1000 kcal*			
Protein (g)	−0.319 (−0.674 to 0.035)	0.100	0.076
Carbohydrates (g)	0.478 (0.120 to 0.836)	0.203	**0.011** *
Fat (g)	−0.345 (−0.731 to 0.041)	0.099	0.078
LCPUFAs (g)	−0.470 (−0.842 to −0.972)	0.185	**0.015** *
Linoleic acid (g)	−0.389 (−0.745 to −0.037)	0.143	**0.034** *
Omega‐6 fatty acids (g)	−0.385 (−0.747 to −0.023)	0.137	**0.038** *
RAE (mcg)	0.552 (0.250 to 0.854)	0.327	**0.001** *
Vitamin E (mg)	−0.310 (−0.668 to 0.049)	0.088	0.088
*Food groups per 1000 kcal*			
Crackers, crispbread (knäckebröd), round toast (g) (*n* = 12)	0.556 (0.245 to0.867)	0.317	**0.001** *
Low fat margarine (g) (*n* = 8)	−0.342 (−0.710 to 0.026)	0.107	0.067

*Note*: Results with *p* < 0.1 are presented.

Abbreviations: g, gram; mcg, microgram; mg, milligram; sIgE, allergen‐specific immunoglobulin E; SPT, Skin Prick Test.

* Significantly related to the outcome, *p* < 0.05.

A trend for a negative association with the threshold level was found for total protein (*β* = –.319, *p* = 0.076), total fat intake (*β* = –.345, *p* = 0.078), vitamin E (*β* = –.310, *p* = 0.088) and low fat margarine (*β* = –.342, *p* = 0.067). Twenty children used supplements of which vitamin D was most frequently used (19 children, 95%). Calculations with or without supplements did not alter our findings. In Supporting Information: Table 3 significant and not‐significant regression analyses are given.

### 3.4 Associations between faeces and saliva microbiota composition, threshold level and outcome

#### 3.4.1 Positive challenges

Faeces and saliva samples were collected in 29 and 28 children, respectively.

No clustering was shown for microbiota composition in faeces or saliva at phylum or species level within the allergic group.

**Figure 1:**
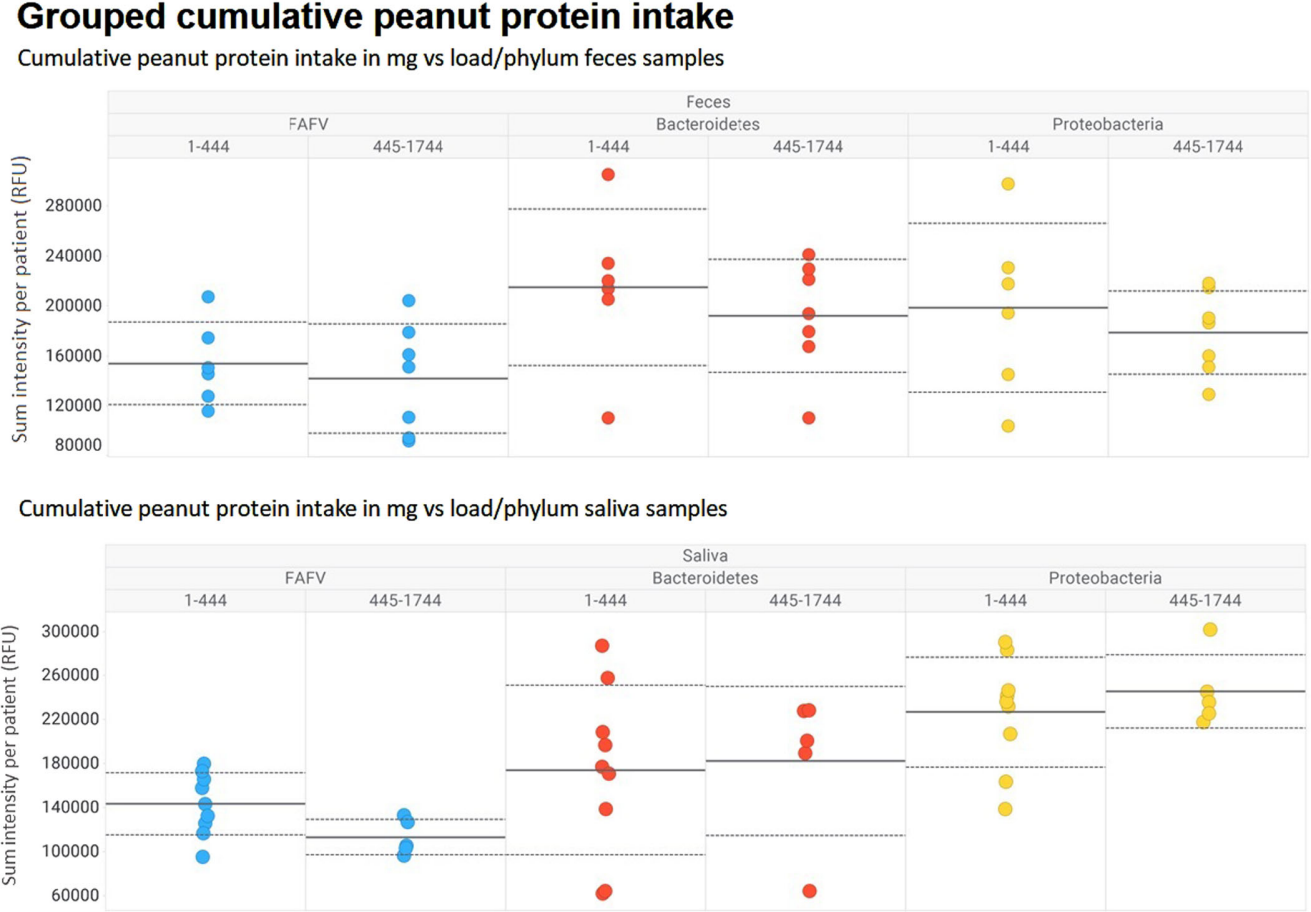
Abundance of saliva and faeces microbiota composition at phylum level according to low (<445 mg peanut protein) or high threshold levels (≥445 mg cumulative peanut protein).

No association was seen between faeces or saliva microbiota composition at phylum level in children with the 50% lowest threshold level (<445 mg peanut protein) and children with the 50% highest threshold doses for peanut (≥445 mg cumulative peanut protein) (Figure 1).

No significant differences in microbiota composition were found for sex, age (younger or older than 3 years of age) or presence of allergic rhinitis or asthma and use of antibiotics. Children without previous use of antibiotics had a higher abundance of Bacteroidetes in both faeces and saliva. Due to small numbers, however, a reliable statistical comparison was not possible.

#### 3.4.2 Positive and negative challenges

**Figure 2:**
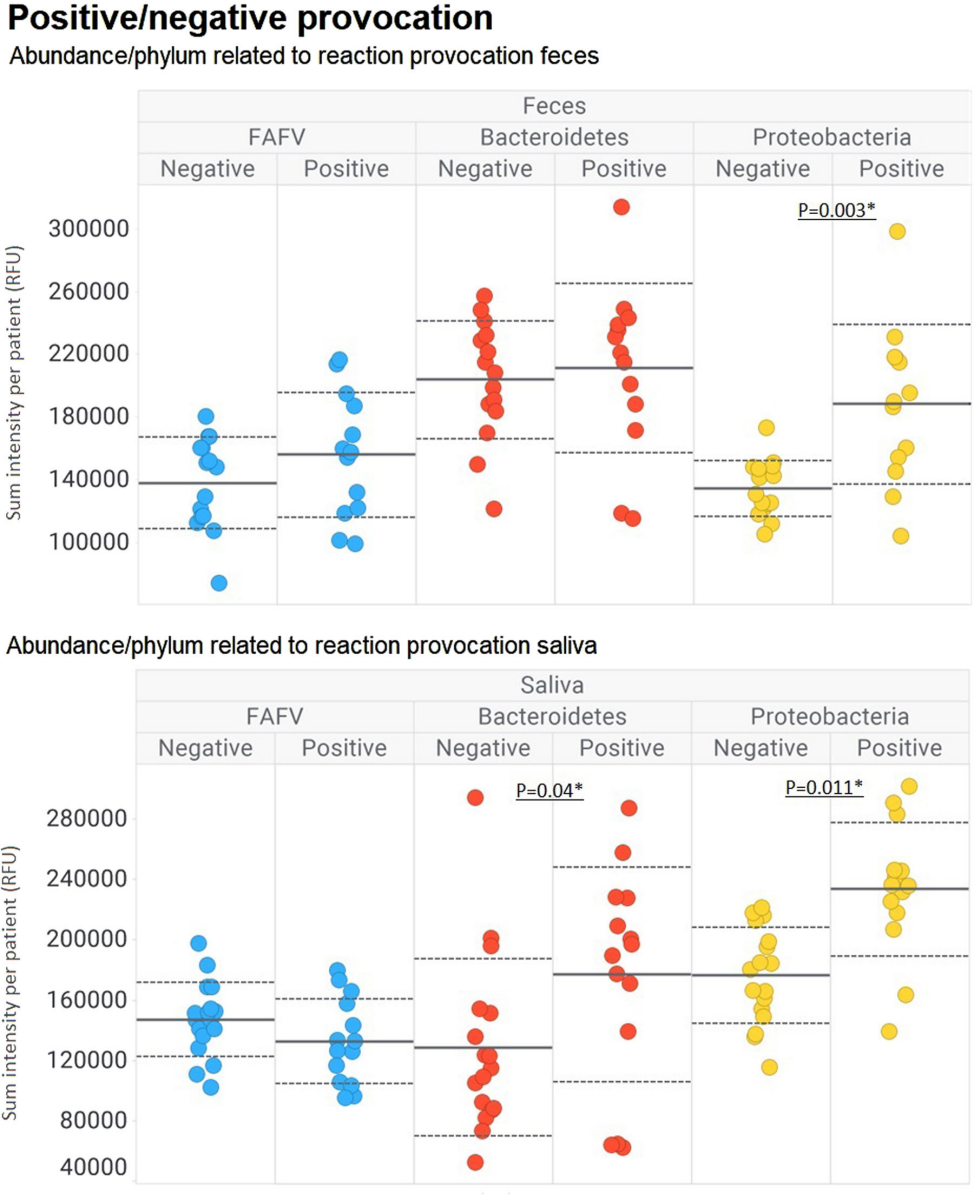
Abundance of saliva and faeces microbiota composition at phylum level according to challenge outcome.

Faecal samples of children with a positive peanut challenge were characterized by a significant higher abundance of Proteobacteria (*n* = 13) children with a negative challenge (*n* = 16), *p* = 0.003 (Figure 2). Saliva samples of children with a positive challenge were found to have significant higher numbers of both Proteobacteria and Bacteroidetes (*n* = 13) compared to the children with a negative challenge (*n* = 15) (*p* = 0.011 and 0.04, respectively) (Figure 2).

## 4 DISCUSSION

In this explorative cross‐sectional study we found that five nutrients (carbohydrates, RAE, LCPUFAs, linoleic acid) and one food group (round toast/crackers) were statistically significant associated with the cumulative threshold level for peanut in peanut‐allergic children. A beneficial association was found for carbohydrates, RAE (vitamin A) and round toast/crackers adjusted for energy intake and senitization. Thus, children with a higher intake of carbohydrates, RAE or round toast/cracker per 1000 kcal had a higher threshold level for peanut.

For LCPUFAs, linoleic acid and omega‐6 fatty acids negative (adverse) associations were found. Thus, children with a higher intake of these fatty acids per 1000 kcal had a lower threshold level for peanut.

Furthermore, we found no association between microbiota composition and peanut threshold, however, significant microbial differences on phylum level, both in saliva and faeces, were observed between allergic and nonallergic children.

The beneficial association of carbohydrates and threshold levels may be explained by the intake of polysaccharides or dietary fibers. Fiber intake leads to the production of SCFAs by the intestinal microbiota. SCFAs play an important role in the maintenance of the intestinal barrier integrity and intestinal epithelium repair.^26^

Vitamin A, for which we found a relationship of its intake with a higher threshold for peanut, is well known for its regulation role in the expression of tight junction proteins on intestinal epithelial cells that are critical for the maintenance of the barrier function in the gut.^27^ Moreover, retinoic acid (vitamin A) imprints the homing of leukocytes to the gut and promotes oral tolerance to food antigens by enhancing the induction of regulatory T‐cells.^28^

Another major finding is that LCPUFAs, linoleic acid, omega‐6 fatty acids from food and low fat margarines were all adversely associated with the threshold level for peanut. The common factor in these variables is linoleic acid, which belongs to the omega‐6 fatty acids. LCPUFAs consist of omega‐6 fatty acids and omega‐3 fatty acids. In the Western diet the largest proportion of LCPUFAs consists of omega‐6 fatty acids, specifically linoleic acid.^29^ The increased intake of omega‐6 fatty acids during the last few decades is linked to the increased prevalence of allergic diseases.^22^ due to its pro‐inflammatory effects.^30^ Linoleic acid is a precursor of arachidonic acid. This fatty acid is involved in the formation of different potent pro‐inflammatory mediators such as leukotrienes and prostaglandins, which may all impact the increased permeability in the gut.^30^

Children with a peanut allergy had a significant abundance of species belonging to the phylum *Proteobacteria*, in both faecal and saliva samples as compared to children who were tolerant to peanut, as well as significantly more *Bacteroidetes* in saliva. Various studies showed that intestinal microbial dysbiosis, including disturbance of *Proteobacteria*, is associated with food allergy.^8^, ^31^

Our finding that microbiota was not significantly associated with threshold levels may be due to a lack of power. Very recently, Zhang et al.^32^ found a higher abundance of *Bacteroides thetaiotaomicron* in stool samples in 13 children with a low threshold level to peanut (<300 mg peanut) as compared to 38 children with a high threshold level to peanut (≥300 mg peanut. In saliva children with a higher threshold had a higher abundance of the commensal *Veillonella nakazawae* than children with a lower threshold.

The strength of our study is that in a novel hypothesis generating approach we studied the associations between the intake of nutrients and foods and threshold levels for food allergens. Dietary composition was taken into account as a novel cofactor when studying threshold levels in patients.

If our data are validated in larger cohorts, this finding will have important clinical consequences for patients: a diet consisting of lower inflammatory components, could increase the threshold levels for peanut and vice versa.

Our study had a few limitations. The data of this study must be interpreted with caution because of the lack of causation and the relatively small sample size. We did not correct for multiple testing, due to the hypothesis generating character of the study. Because of the small sample size we limited our analyses and, for example, did not determine short chain fatty acids (SCFAs) in stools. Lastly, we also did not ascertain for our hypothesis that nutrition impacts on barrier function and that an increased gut permeability is associated with decreased threshold levels. This hypothesis is currently studied in a randomized controlled trial in children with peanut and/or nut allergy, ClinicalTrials.gov, NCT05667610.

In conclusion, as a novel concept, we showed that dietary composition is related to threshold levels for peanut. Increasing threshold levels for peanut by diet may be a promising approach.

## 5 IMPACT STATEMENT

If confirmed in larger studies dietary composition may either increase or decrease threshold levels for peanut, which may have important clinical consequences for patients. In addition, dietary composition may have to be considered as cofactor in threshold studies.

## AUTHOR CONTRIBUTIONS

**Daisy Luiten**: Formal analysis; investigation; methodology; project administration; resources; writing—original draft. **Maarten Biezeveld**: Data curation; investigation; methodology; resources; writing—original draft. **Olga van Doorn**: Data curation; investigation; methodology; resources; writing—original draft. **Hanae Riady**: Data curation; formal analysis; investigation; methodology; resources; writing—original draft. **Ming Yang**: Data curation; Formal analysis; investigation; methodology; resources; writing—original draft. **Femke Bergsma**: Data curation; investigation; methodology; resources; writing—original draft. **Atie van der Plas**: Data curation; investigation; methodology; resources; writing—original draft. **Kim Brand**: Data curation; investigation; methodology; resources; writing—original draft. **Nicolette Arends**: Data curation; investigation; methodology; resources; writing—original draft. **Annette de Bruin**: Data curation; investigation; methodology; resources; writing—original draft. **Jeanne de Vries**: Data curation; investigation; methodology; resources; writing—original draft. **Tim de Meij**: Data curation; formal analysis; investigation; methodology; resources; writing—original draft. **Berber Vlieg‐Boerstra**: Conceptualization; data curation; formal analysis; investigation; methodology; resources; supervision; writing—original draft; writing—review & editing.

## CONFLICTS OF INTEREST STATEMENT

Vlieg–Boerstra received research funding from Nutricia, consulting or speaker's fees from Marfo Food groups, Nestlé, Abbott and Nutricia. The remaining authors declare no conflict of interest.

## Supporting information

Supporting information.

Supporting information.

Supporting information.

Supporting information.

## Data Availability

The data that support our findings can be shared on request from the corresponding author.
