# Supplementary material for: Peanut thresholds in peanut‐allergic children are related to dietary composition
Source: Immun Inflamm Dis. 2023 May 26;11(5):e841. doi: 10.1002/iid3.841 (PMC10214579; doi:10.1002/iid3.841)
Supplement: Supplementary file 1 — Supporting information. [file IID3-11-e841-s003.docx]

**Electronic Repository. Appendix.**

**IS-pro technique**

IS-pro is a DNA-based microbiota profiling technique, based on the identification of species-specific

length polymorphisms of the interspacer (IS) region and phylum-specific sequence

polymorphisms of 16S rDNA. In short, 300 μl lysis buffer is added to an Eppendorf container

containing 0.5 gr frozen faeces or saliva sample. This mixture is vortexed and subsequently shaken for 5 minutes at room temperature. The containers are centrifuged for 2 minutes at 13,000g. The

resulting supernatant is subsequently transferred to an empty DNA isolation vial, followed

by adding magnetic silica beads according to the routine protocol. Bacterial DNA is isolated

by a standard automated isolation procedure (EasyMag, Biomereux, Marcy l’Etoile, France).

The resulting total DNA is eluted in 110 μl buffer and stored at 4°C until use for PCR amplification.

ISpro technique: Isolated DNA (10μl/PCR) is amplified in two normal standardized

multiplex PCR amplifications: (1) Firmicutes, Actinobacteria, Fusobacteria, Verrucomicrobia

(FAFV), Bacteroidetes and (2) Proteobacteria. The forward FAFV, Bacteriodetes and Proteobacteria

primers contain different fluorescent labels for phylum identification.

After amplification, 5 μl of PCR product is mixed with 20 μl formamide and 0,2 μlMapmaker 1500 ROX labeled sizemarker (custom made by BioVentures, Murfreesboro, TN, USA). Subsequently, PCR products are separated based on their different lengths in an ABI Prism 3130XL Genetic Fragment

Analyzer (Applied Biosystems Carlsbad, California, USA). Overall, three levels of information

are obtained: color of peaks discriminates detected peaks into the phyla FAFV, Bacteroidetes

and Proteobacteria, together covering the major phyla present in the human gut. Measured

length of the 16S–23S rDNA IS region, displayed by number of nucleotides, is used to identify

bacteria at both pylum and species level based on a database consisting of more than 1500 species and their corresponding IS lengths. Peak height, measured in relative fluorescence units (RFU), corresponds to the quantity of PCR product, reflecting the relative abundance of present species.

Details about data analysis using IsPro software are described by Buddinget al.^35^
