# Supplementary material for: Peanut thresholds in peanut‐allergic children are related to dietary composition
Source: Immun Inflamm Dis. 2023 May 26;11(5):e841. doi: 10.1002/iid3.841 (PMC10214579; doi:10.1002/iid3.841)
Supplement: Supplementary file 2 — Supporting information. [file IID3-11-e841-s001.docx]

**Electronic Repository Table I.** Overview of nutrients analysed in this study.

| Nutrients |
| --- |
| Total protein |
| Protein energy % (energy%) |
| Total fat |
| Fat eergyn% |
| Saturated fat |
| Polyunsaturated fatty acids (LCPUFAs) |
| Monounsaturated fatty acids (MUFAs) |
| α-linoleic acid (ALA) |
| Eicosapentaenoic acid (EPA) |
| Docosahexaenoic acid (DHA) |
| n-6 LCPUFAs |
| n-3 LCPUFAs |
| n6:n3 ratio |
| Total carbohydrates |
| Carbohydrates energy% |
| Mono- and disaccharides |
| Polysaccharides |
| Dietary fibres |
| Dietary fibres energy% |
| Alcohol |
| Alcohol energy% |
| Calcium |
| Phosphorus |
| Iron |
| Sodium* |
| Potassium |
| Magnesium |
| Zinc |
| Selenium |
| Copper |
| Iodine |
| Retinol |
| Vitamin B1 |
| Vitamin B2 |
| Vitamin B3 |
| Vitamin B6 |
| Vitamin B12 |
| Vitamin D |
| Vitamin E |
| Vitamin C |
| Folate equivalent (natural B9 in food) |
| Retinol Activity Equivalent (RAE) |

*The intake of added salt was not calculated
