# Supplementary material for: Peanut thresholds in peanut‐allergic children are related to dietary composition
Source: Immun Inflamm Dis. 2023 May 26;11(5):e841. doi: 10.1002/iid3.841 (PMC10214579; doi:10.1002/iid3.841)
Supplement: Supplementary file 3 — Supporting information. [file IID3-11-e841-s002.docx]

**Electronic Repository Table II.** The division of the food groups.

| Main food groups | Sub-food group level 1 | Sub-food group level 2 |
| --- | --- | --- |
| Potatoes (1) |  |  |
|  | Cooked potatoes and mashed potatoes (1.1) |  |
|  | Fries and baked potatoes (1.2) |  |
|  | Sweet potato and cassava (1.3) |  |
| Alcoholic and non-alcoholic drinks (2) |  |  |
|  | Coffee/tea (2.1) |  |
|  | Processed fruit juice and vegetable juice (2.2) |  |
|  | Ready-to-use breakfast drink  (2.3) |  |
|  | Soda/lemonade/sport drinks (2.4) |  |
|  | Water (2.5) |  |
|  | Beer (2.6) |  |
|  | Wine (2.7) |  |
|  | Liquor (2.8) |  |
| Bread and crackers (3) |  |  |
|  | Crackers, crispbread, round toast(3.1) |  |
|  | Bread, rye bread (3.2) |  |
|  |  | Whole grain bread (3.2.1) |
|  |  | White bread (3.2.2) |
|  |  | Brown and multigrain bread (3.2.3) |
|  |  | Croissants (3.2.4) |
|  |  | Bread with raisins/almond paste (3.2.5) |
| Various products (4) |  |  |
|  | Sweeteners ( 4.1) |  |
|  | Diverse ( 4.2) |  |
| Eggs (5) |  |  |
| Fruit (6) |  |  |
|  | Fresh fruit (6.1) |  |
|  | Apple sauce, fruit conservatives  (6.2) |  |
|  | Dried fruit (6.3) |  |
|  | Fresh fruit juice (6.4) |  |
| Pastries and cake (7) |  |  |
|  | Small cookies and biscuits (7.1) |  |
|  | Cake and large cookies (7.2) |  |
|  | Pastries and pie (7.3) |  |
|  | Gingerbread ( 7.4) |  |
|  | Muesli bars/Meal bars ( 7.5) |  |
| Grain products (8) |  |  |
|  | Breakfast cereals without oats (8.1) |  |
|  | Whole grain cereals and bonding agents (8.2) |  |
|  | Refined grains and bonding agents (8.3) |  |
|  | Other grains and boding agents (8,4) |  |
| Vegetables (9) |  |  |
|  | Cookedvegetables (9.1) |  |
|  | Raw vegetables/fresh olives (9.2) |  |
|  | Fresh vegetable juice (9.3) |  |
| Savoury spreads/Nut butters (10) |  |  |
|  | Peanut butter, nut butter and seed butter (10.1) |  |
|  | Vegetable spread (10.2) |  |
|  | Tapenade, pesto and sun-dried tomato (10.3) |  |
| Cheese products (11) |  |  |
|  | Cheese, fresh cheese, semi-skimmed and full fat curd (11.1) |  |
|  | Cheese spread and other cheese products (11.2) |  |
|  | Low fat cheese (<40% fat), skimmed curd (11.3) |  |
| Herbs and spices (12) |  |  |
| Milk and dairy products (13) |  |  |
|  | Fresh milk and milk drinks (13.2) |  |
|  |  | Skimmed milk (13.2.1) |
|  |  | Semi-skimmed and full fat milk (13.2.2) |
|  | Dairy drinks and hot chocolate milk (13.3) |  |
|  | Ice cream and milkshakes (13.4) |  |
|  | Condensed milk (13.5) |  |
|  | Cream (13.6) |  |
|  | Fermented dairy (13.7) |  |
|  |  | Skimmed fermented dairy (13.7.1) |
|  |  | Semi-skimmed and full fat fermented dairy (13.7.2) |
|  | Custard and porridge (13.8) |  |
|  |  | Whole grain porridge (13.8.1) |
|  | Toppings (13.9) |  |
| Soy products and vegetarian products (14) |  |  |
|  | Soy milk, soy dessert, soy drink and soy cream (14.1) |  |
|  |  | Plant-based yoghurt (14.4.1) |
|  | Vegetarian sliced meat (14.2) |  |
|  | Vegetarian meat substitutes (14.3) |  |
|  | Other soy products (14.4) |  |
|  | Plant based drinks other than soy (rice-, haver-, almond or coconut milk) (14.5) |  |
| Nuts, seeds and snacks (15) |  |  |
|  | Nuts/seeds (15.1) |  |
|  |  | Peanuts (15.1.1) |
|  | Chips/pretzels (15.2) |  |
|  | Savory snacks (15.3) |  |
|  | Salads for bread and toast (15.4) |  |
| Legume/hummus (16) |  |  |
| Nutritional preparations (17) |  |  |
|  | Meal substitutes (17.1) |  |
|  | Sport supplements) (17.2) |  |
|  | Formulas (17.3) |  |
| Ready meals (18) |  |  |
|  | Pancake, pizza (18.1) |  |
|  | Ready meals (18.2) |  |
|  |  |  |
| Soups (19) |  |  |
|  | Soups with legumes (19.1) |  |
|  | Soups without legumes (19.2) |  |
| Sugar, sweets, sweet spread and sweet sauces (20) |  |  |
|  | Chocolate, bonbons and candy bars (20.1) |  |
|  | Candy and sweets (20.2) |  |
|  | Sugar (20.3) |  |
|  | Sweet toppings and spreads (20.4) |  |
| Fats, oils and savory sauces (21) |  |  |
|  | Butter (21.1) |  |
|  | Low-fat margarine (21.2) |  |
|  |  | Low-fat margarine with omega-3 (21.2.1) |
|  | Margarine/liquid margarine/cooking oil (21.3) |  |
|  |  | Margarine/liquid margarine/cooking oil with omega-3 (21.3.1) |
|  | Oil (21.4) |  |
|  |  | Olive oil (21.4.1) |
|  |  | Omega-6 rich oil (21.4.2) |
|  |  | Omega-3 rich oil (21.4.3) |
|  | Frying fat (21.5) |  |
|  | Ready-made sauce and gravy (21.6) |  |
| Fish products (22) |  |  |
|  | Lean fish (22.1) |  |
|  | Semi-fat fish (22.2) |  |
|  | Fatty fish (22.3) |  |
|  | Fried fish (22.4) |  |
|  | Shellfish (22.5) |  |
| Meat products (23) |  |  |
|  | Minced meat (23.1) |  |
|  | Chicken and poultry (23.2) |  |
|  | Red meat (23.3) |  |
|  | Meat products (23.4) |  |
|  | Liver products (23.5) |  |

*Food groups classification as defined in Compleat was used, which in turn was based on the Dutch National Food Consumption Survey ^34^ In addition some additional subgroups were made for methods of processing, fibre or fat content and fermentation, such as fresh and processed fruits and fruit products (e.g. prepacked fruit juiced), raw and cooked vegetables, whole grain bread and grains, fermented dairy, skimmed, semi skimmed and full fat dairy and cheese, peanuts and nuts, butter, omega-6 and omega-3 rich oil, lean, semi-fat and fatty fish.*

*The intake of water (2.5) and herbs and spices (12) was not recorded.*
