# Supplementary material for: Peanut thresholds in peanut‐allergic children are related to dietary composition
Source: Immun Inflamm Dis. 2023 May 26;11(5):e841. doi: 10.1002/iid3.841 (PMC10214579; doi:10.1002/iid3.841)
Supplement: Supplementary file 4 — Supporting information. [file IID3-11-e841-s004.docx]

| **Electronic Repository Table III.** Complete list of associations in peanut-allergic children (n=32) between intake of nutrients and foods and cumulative threshold levels in milligram peanut protein adjusted for sensitisation assessed by multivariable linear regression analyses | **№ of consumers** | **Standardized β Coefficients (95% CI),  adjusted for sIgE and SPT categories** | **Adjusted R^2^** | **P-value** |
| --- | --- | --- | --- | --- |
| **Nutrients per 1000 kcal** |  |  |  |  |
| Protein (g) | 32 | -0.319 (-0.674 to 0.035) | 0.100 | 0.076 |
| Carbohydrates (g) | 32 | 0.478 (0.120 to 0.836) | 0.203 | **0.011*** |
| Mono- and disaccharides (g) | 32 | 0.243 (-1.16 to 0.603) | 0.056 | 0.176 |
| Polysaccharides (g) | 32 | 0.177 (-2.15 to 0.570) | 0.021 | 0.363 |
| Fat (g) | 32 | -0.345 (-0.731 to 0.041) | 0.099 | 0.078 |
| Saturated fat (g) | 32 | -0.175 (-0.556 to 0.206) | 0.022 | 0.354 |
| Mono Unsaturated fat (g) | 32 | -0.142 (-0.540 to 0.257) | 0.010 | 0.473 |
| LCPUFAs (g) | 32 | -0.470 (-0.842 to -0.972) | 0.185 | **0.015*** |
| Linoleic acid (g) | 32 | -0.389 (-0.745 to -0.037) | 0.143 | **0.034*** |
| Omega-3 fatty acids (g) | 32 | -0.127 (-0.498 to 0.243) | 0.008 | 0.487 |
| Omega-6 fatty acids (g) | 32 | -0.385 (-0.747 to -0.023) | 0.137 | **0.038*** |
| omega6.omega3.ratio | 32 | -0.053 (-0.475 to 0.364) | 0.036 | 0.787 |
| ALA (g) | 32 | -0.205 (-0.568 to 0.158) | 0.037 | 0.257 |
| EPA (g) | 32 | -0.078 (-0.461 to 0.304) | -0.003 | 0.677 |
| DHA (g) | 32 | -0.084 (-0.468 to 0.300) | -0.002 | 0.656 |
| Dietary fiber (g) | 32 | -0.070 (-0.444 to 0.304) | -0.004 | 0.704 |
| Calcium (mg) | 32 | -0.121 (-0.506 to 0.263) | 0.006 | 0.524 |
| Phosphorus (mg) | 32 | -0.225 (-0.594 to 0.143) | 0.044 | 0.220 |
| Iron (mg) | 32 | -0.109 (-0.506 to 0.288) | 0.002 | 0.577 |
| Magnesium (mg) | 32 | -0.098 (-0.468 to 0.273) | 0.001 | 0.594 |
| Zinc (mg) | 32 | -0.279 (-0.639 to 0.081) | 0.074 | 0.124 |
| Selenium (mcg) | 32 | -0.213 (-0.578 to 0.152) | 0.040 | 0.243 |
| Copper (mg) | 32 | -0.69 (-0.490 to 0.353) | -0.005 | 0.741 |
| Iodine (mcg) | 32 | -0.097 (-0.512 to 0.319) | -0.001 | 0.638 |
| RAE (mcg) | 32 | 0.552 (0.250 to 0.854) | 0.327 | **0.001*** |
| Vitamin B1 (mg) | 32 | -0.238 (-0.605 to 0.130) | 0.050 | 0.196 |
| Vitamin B2 (mg) | 32 | -0.124 (-0.519 to 0.271) | 0.006 | 0.524 |
| Vitamin B3 (mg) | 32 | -0.098 (-0.467 to 0.272) | 0.001 | 0.593 |
| Vitamin B6 (mg) | 32 | -0.031 (-0.406 to 0.345) | -0.008 | 0.868 |
| Vitamin B11 (mcg) | 32 | 0.096 (-0.282 to 0.474) | 0.000 | 0.608 |
| Vitamin B12 (mcg) | 32 | -0.096 (-0.476 to 0.284) | 0.000 | 0.609 |
| Vitamin C (mg) | 32 | 0.150 (-0.219 to 0.519) | 0.015 | 0.413 |
| Vitamin D (mcg) | 32 | -0.117 (-0.499 to 0.266) | 0.005 | 0.538 |
| Vitamin E | 32 | -0.310 (0.668 to 0.049) | 0.092 | 0.088 |
|  |  |  |  |  |
| **Foods or food groups per 1000 kcal in gram (39 food groups)** |  |  |  |  |
| Cooked potatoes and mashed potatoes (1.1) | 19 | 0.160 (-0.206 to 0.525) | 0.019 | 0.378 |
| Fries and baked potatoes (1.2) | 8 | 0.019 (-0.359 to 0.398) | 0.061 | 0.917 |
| Coffe/tea (2.1) | 9 | -0.081 (-0.451 to 0.288) | -0.002 | 0.655 |
| Processed fruit juice and vegetable juice (2.2) | 16 | -0.128 (-0.504 to 0.248) | 0.008 | 0.491 |
| Soda/lemonade/sport drinks (2.4) | 21 | -0.050 (-0.433 to 0.332) | -0.007 | 0.789 |
| Crackers, crispbread (knäckebröd), round toast (3.1) | 12 | 0.556 (0.245 to 0.867) | 0.317 | **0.001** |
| whole grain bread (3.2.1) | 21 | -0.155 (-0.523 to 0.213) | 0.017 | 0.395 |
| white bread (3.2.2) | 18 | -0.102 (-0.518 to 0.314) | 0.000 | 0.619 |
| Brown and multigrain bread (3.2.3) | 13 | 0.267 (-0.118 to 0.652) | 0.059 | 0.166 |
| Bread with raisins/almond paste (3.2.5) | 8 | 0.018 (-0.316 to 0.395) | -0.009 | 0.924 |
| Fresh fruit( 6.5) | 30 | 0.257 (-0.119 to 0.633) | 0.057 | 0.174 |
| Apple sauce, fruit conservatives (6.2) | 12 | 0.126 (-0.242 to 0.495) | 0.008 | 0.488 |
| Dried fruit (6.3) | 11 | 0.138 (-0.233 to 0.509) | 0.011 | 0.453 |
| Small cookies and biscuits (7.2) | 20 | -0.100 (-0.391 to 0.372) | -0.009 | 0.959 |
| Cake and large cookies (7.3) | 12 | 0.084 ((-0.258 to 0.453) | -0.001 | 0.466 |
| Breakfast cereals wihout oats (8.1) | 11 | -0.026 (-0.402 to 0.350) | -0.008 | 0.888 |
| Whole grain cereals and bonding agents (8.2) | 26 | 0.092 (-0.283 to 0.467) | 0.000 | 0.619 |
| Cooked vegetables (9.1) | 27 | -0.066 (-0.457 to 0.325) | -0.005 | 0.731 |
| Raw vegetables/fresh olives (9.2) | 27 | 0.016 (-0.375 to 0.407) | -0.009 | 0.935 |
| Cheese, fresh cheese, semi skimmed and full fat curd (11.1) | 21 | 0.253 (-0.111 to 0.617) | 0.059 | 0.165 |
| Semi-skimmed and full fat milk (13.2.2) | 19 | 0.019 (-0.302 to 0.335) | -0.009 | 0.918 |
| Ice cream and milkshakes (13.4) | 8 | 0.060 (--0.310 to 0.431) | -0.005 | 0.741 |
| Fermented dairy (13.7) | 9 | -0.076 (-0.453 to 0.301) | -0.003 | 0.683 |
| Chips/pretzels (15.2) | 7 | 0.082 (-0.295 to 0.459) | -0.002 | 0.660 |
| Soups without legumes (19.2) | 9 | -0.076 (-0.481 to 0.329) | -0.004 | 0.703 |
| Chocolade. Bonbons and candybars (20.1) | 13 | -0.139 (-0.500 to 0.231) | 0.012 | 0.447 |
| Candy and sweets (20.2) | 11 | -0.206 (-0.569 to 0.157) | 0.037 | 0.254 |
| Sugar (20.3) | 8 | -0.119 (-0.491 to 0.252) | 0.006 | 0.516 |
| Sweet toppings and spreads (20.4) | 25 | -0.108 (-0.494 to 0.279) | 0.002 | 0.572 |
| Butter (21.1) | 15 | -0.105 (-0.476 to 0.266) | 0.003 | 0.566 |
| Low-fat margarine (21.2) | 8 | -0.342 (-0.710 to 0.026) | 0.107 | **0.067** |
| Margarine/lliquid margarine/cooking oil (21.3) | 12 | 0.380 (-0.349 to 0.425) | -0.008 | 0.844 |
| Olive oil (21.4.1) | 18 | 0.124 (-0.253 to 0.502) | 0.007 | 0.505 |
| Omega-6 rich oil (21.4.2) | 7 | -0.226 (-0.613 to 0.161) | 0.040 | 0.241 |
| Fatty fish (22.3) | 10 | 0.037 (-0.337 to 0.411) | -0.008 | 0.840 |
| Minced meat (23.1) | 9 | 0.283 (-0.880 to 0.653) | 0.072 | 0.13 |
| Chicken and poultry (23.2) | 11 | -0.228 (-0.599 to 0.143) | 0.045 | 0.219 |
| Read meat (23.3) | 14 | -0.082 (-0.456 to 0.292) | -0.002 | 0.675 |
| Meat products (23.4) | 20 | -0.063 (-0.455 to 0.329) | -0.005 | 0.745 |
|  |  |  |  |  |

significantly related to the outcome, p < 0.05
g, gram; mcg, microgram; mg, milligram; sIgE, allergen-specific immunoglobulin E; SPT, Skin Prick Test.
